# Changing prevalence of darunavir resistance-associated mutations (DRV RAMs) in clinical samples received for routine resistance testing: 2003-2009

**DOI:** 10.1186/1758-2652-13-S4-P132

**Published:** 2010-11-08

**Authors:** G De La Rosa, T Pattery, G Picchio, E Lathouwers, J Villacian, K Van der Borght

**Affiliations:** 1 Tibotec Therapeutics, 1125 Trenton-Harbourton Road, Titusville, NJ, USA; 2 Virco BVBA, Mechelen, Belgium; 3 Tibotec Therapeutics, Titusville, NJ, USA; 4 Tibotec BVBA, Beerse, Belgium

## Purpose of the study

Darunavir (DRV) is an HIV protease inhibitor (PI) first approved in 2006. This analysis evaluated the prevalence of DRV resistance associated mutations (RAMs) in clinical samples submitted for routine resistance testing to assess potential changes or evolution in the frequency of these mutations over time.

## Methods

Annual prevalence of the IAS-USA 2009 DRV RAMs (V11I, V32I, L33F, I47V, I50V, I54L/M, T74P, L76V, I84V, and L89V) was studied in approximately 232,000 routine clinical samples submitted to Virco for resistance testing between Jan 2003 and Dec 2009. Prevalence was assessed over time for individual DRV RAMs, DRV RAM combinations and presence of 0, 1, 2, or ≥3 DRV RAMs. Results for DRV RAMs were expressed as the proportion of (1) all clinical samples, (2) samples with evidence of PI resistance (defined by FDA mutation list, or a predicted fold change (FC) in IC50 for any PI greater than the respective virco®TYPE HIV-1 (VTY) lower clinical cut-off [CCO] (FC=10) and (3) samples with DRV resistance defined by predicted FC >10.

## Summary of results

**Figure 1:**
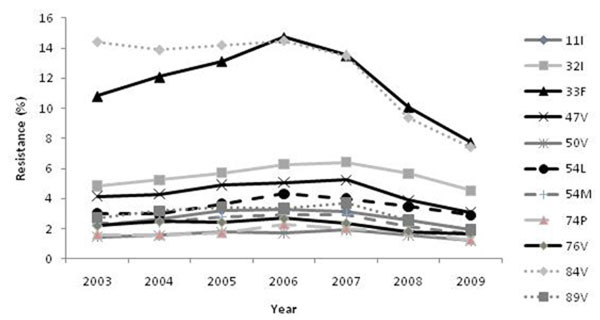
2009 IAS-USA DRV RAMs as a proportion of samples with evidence of PI resistance by VTY lower CCO.

Overall prevalence of samples showing evidence of any PI resistance decreased gradually over time (from 2003 to 2009: 47.0% to 32.2% [VTY lower CCO]; 49.1% to 42.2% [US-FDA mutation list]. Mean prevalence of each of the 11 individual DRV RAMs also decreased over time (Figure 1)

Prevalence of samples harbouring ≥1 DRV RAMs also decreased over time. In 2009, 94.3% of all samples harboured zero DRV RAMs versus 85.3% in 2003. Among samples with evidence of PI resistance, 88% vs 72% (per FDA list) and 84% vs 70% (to any PI defined by predicted FC> low CCO) harboured zero DRV RAMs. The most common three DRV RAM combination was L33F,I54L,I84V which was detected with a prevalence of 0.15% in 2003 and 0.08% in 2009.

## Conclusions

In 2009, most routine clinical HIV isolates (94.3%) harboured zero DRV RAMs. Despite widespread DRV use, the prevalence of DRV RAMs among all clinical isolates and among those with evidence of PI resistance has decreased since 2003. This could be due to pharmacologic suppression on the mutation rate and/or DRV's high genetic barrier to the development of resistance within the treatment regimen.

